# Review: Synthetic scaffolds to control the biochemical, mechanical, and geometrical environment of stem cell-derived brain organoids

**DOI:** 10.1063/1.5045124

**Published:** 2018-11-15

**Authors:** Mariana Oksdath, Sally L. Perrin, Cedric Bardy, Emily F. Hilder, Cole A. DeForest, R. Dario Arrua, Guillermo A. Gomez

**Affiliations:** 1 Centre for Cancer Biology, South Australia Pathology and University of South Australia, Adelaide 5001, Australia; 2 South Australian Health and Medical Research Institute (SAHMRI), Adelaide 5000, Australia; 3 Flinders University, College of Medicine and Public Health, Bedford Park 5042, Australia; 4 Future Industries Institute, University of South Australia, Mawson Lakes 5095, Australia; 5 Department of Chemical Engineering and Department of Bioengineering, University of Washington, Seattle, Washington 98195-1750, USA

## Abstract

Stem cell-derived brain organoids provide a powerful platform for systematic studies of tissue functional architecture and the development of personalized therapies. Here, we review key advances at the interface of soft matter and stem cell biology on synthetic alternatives to extracellular matrices. We emphasize recent biomaterial-based strategies that have been proven advantageous towards optimizing organoid growth and controlling the geometrical, biomechanical, and biochemical properties of the organoid's three-dimensional environment. We highlight systems that have the potential to increase the translational value of region-specific brain organoid models suitable for different types of manipulations and high-throughput applications.

## INTRODUCTION

Recent advances in stem cell (SC) and developmental biology have propelled the design of three-dimensional (3D) brain organoids (Lancaster *et al.*, 2013). Brain organoids reflect human brain tissue during early embryogenesis and allow scientists to investigate the interactions between different cell types within the developing brain and the self-organization processes that lead to the functional tissue cytoarchitecture. Moreover, these methodologies demonstrate great promise in the development of personalized therapies in patients affected by different types of brain diseases including microcephalia, Alzheimer's disease (AD), and brain cancers (Hubert *et al.*, 2016; Qian *et al.*, 2016; and Raja *et al.*, 2016).

Although brain organoids grow in a 3D environment, the exact nature by which 3D-presented biochemical, biomechanical, and geometrical signals contribute to brain organoid development is poorly understood. This current limitation emerges from the inability to manipulate the organoids' environment and the lack of knowledge and actual measurements of tissue biomechanical properties in the developing human brain. Here, we review key recent advances in these areas, including the characterization of the mechanical properties of brain tissues [using atomic force microscopy (AFM) and magnetic resonance imaging (MRI)] and the molecular composition of the extracellular matrix (ECM). Understanding the brain microenvironment is important in designing synthetic matrices that mimic anatomically correct human brain tissue. We also review how new synthetic hydrogels that reproduce the 3D microenvironment during organ morphogenesis help to generate organoids with less variability and higher reproducibility. Although these synthetic matrices have not yet been extensively applied to engineer brain organoids, we will discuss how they can be used to better understand key processes that occur during brain development. Finally, we will consider the applications of these synthetic matrices in modelling brain disease and their potential in high-throughput applications.

## ORGANOID MODELS OF HUMAN BRAIN DISEASES

**FIG. 1.**
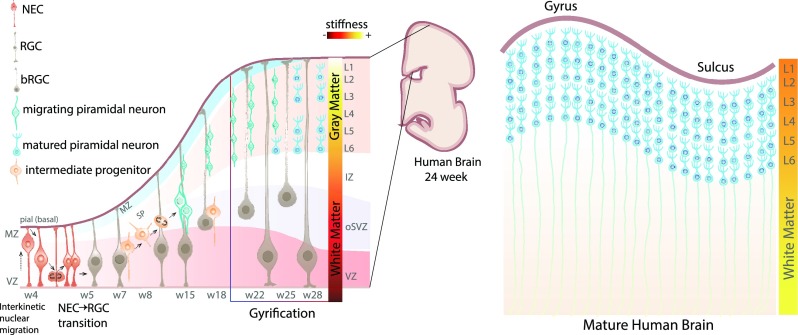
Schematic of human brain development. (Left) Representation of early brain cortical development. During the first weeks of gestation, the neuroepithelium expands by the symmetric division of neuroepithelial stem cells (NECs). During this phase, cells exhibit interkinetic nuclear migration. These processes occur in the ventricular zone (VZ) of the developing neural tube. Neurogenesis begins at week 5 (w5), where radial glial cells (RGCs) experience asymmetric cell division, by which one of the daughter cells will give rise to intermediate progenitors (IPs). Later, IPs symmetrically divide to generate two pyramidal neurons by week 8 (w8). At later stages, where the gyrification begins (week 22, w22), the mechanical properties across different cortical regions change and the white matter becomes softer than the grey matter. (Right) Simplified illustration of a mature human brain where there have been reported differences in stiffness between white matter and grey matter, but this really depends on the technique that was used for measurements and the brain developmental stage. NEC, neuroepithelial stem cell; RGC, radial glial cell; bRGC, basal radial glial cell; VZ: ventricular zone; MZ: marginal zone; SP: subplate; oSVZ: outer subventricular zone; IZ: intermediate zone; and L1-L6: neuronal layers 1 to 6.

Brain diseases such as autism, cortical development malformations, neurodegenerative diseases, and brain cancers have had relatively little progress in their treatment due to a lack of experimental models that permit their study in a physiologically relevant setting (Northcott, 2017 and Simeonova and Huillard, 2014). Brain tissues are remarkably complex functional structures that originate primarily in development and whose cytoarchitecture allows the establishment of neuronal circuits that are central in human cognition (Bota *et al.*, 2015; Crouch *et al.*, 2018; and Wang and Liu, 2014). This is exemplified in the neocortex, which is composed of a variety of different cell types that originates from a single pseudostratified layer of neuroepithelial stem cells (NSCs) through a series of developmental and genetic processes leading to the formation of a multilayered tissue structure (Fig. 1, Kelava and Lancaster, 2016 and Taverna *et al.*, 2014).

These complex brain structures permit different cell types to establish specific interactions between them and with the ECM, creating a variety of different niches with defined biochemical and biomechanical properties that profoundly influence cell proliferation, survival, and differentiation (Barnes *et al.*, 2017; Frith *et al.*, 2018; Guo *et al.*, 2014; Przybyla *et al.*, 2016; Smith *et al.*, 2018; and Yang *et al.*, 2016). The biophysical and biochemical properties of the microenvironment that instruct cells, in space and time, to form fully functional integrated tissues, are poorly characterized (Barnes *et al.*, 2017). A better understanding of how the cytoarchitecture of the brain is developed and affected by disease requires experimental models that (i) recapitulate human brain tissue structures in 3D and (ii) permit direct and independent manipulation of their mechanical and biochemical properties, both of which have not been possible with current experimental approaches such as animal models and monotypic culture of cell lines.

Recently, brain organoids emerged as promising models to better understand brain development and the mechanisms that contribute to the pathology of brain diseases and constitute a powerful pre-clinical model for the development of personalized treatment strategies (Lancaster and Knoblich, 2014b and Pasca, 2018). Brain organoids are derived from embryonic cells (ESCs) or induced pluripotent stem cells (iPSCs) and composed of different cell populations that self-organize forming cortical structures that resemble the human cortex early in development (Camp *et al.*, 2015; Lancaster *et al.*, 2013; Luo *et al.*, 2016; and Renner *et al.*, 2017). To date, brain organoid protocols have been successfully implemented to grow brain organoids that correspond to different brain regions including the dorsal and the ventral cortex (Lancaster *et al.*, 2013 and Xiang *et al.*, 2017), hindbrain and midbrain (Birey *et al.*, 2017; Jo *et al.*, 2016; Lancaster *et al.*, 2013; Monzel *et al.*, 2017; Qian *et al.*, 2016; Quadrato *et al.*, 2017; and Renner *et al.*, 2017), cerebellum (Muguruma *et al.*, 2015), hypothalamus (Qian *et al.*, 2016) and hippocampus (Sakaguchi *et al.*, 2015) and exhibit the capacity to establish discrete neural circuits between different regions (Quadrato *et al.*, 2017). Still, the most powerful concept behind stem cells and organoid technology is that, in principle, it is possible to obtain brain disease organoids from patient-derived iPSCs (Di Lullo and Kriegstein, 2017; Hubert *et al.*, 2016; Ilieva *et al.*, 2018; and Qian *et al.*, 2016).

Recently, brain organoid technology was used to respond to the ZIKA virus global health emergency. Qian *et al.* showed the influence of ZIKA virus at various stages of human embryogenesis in brain organoids (Qian *et al.*, 2016). This research revealed that organoids infected with ZIKA virus show impairment of neural progenitor cells (NPS) and exhibit greater cortical malformation. Hubert *et al.* developed a physiologically relevant patient-derived tumor-organoid model, allowing the assessment of cancer cell invasion (Hubert *et al.*, 2016). This research showed that non-stem cells within organoids were sensitive to radiotherapy, stopping the cell division. Moreover, this developing technology can also resemble patient tissue that can be used to better understand the patient's specific pathological conditions. For example, Raja *et al.* developed a 3D brain organoid model of Alzheimer's Disease (AD) using iPSCs derived from AD patients (Raja *et al.*, 2016). They found that the resulting organoids recapitulated different AD-pathologies including amyloid aggregation, hyper-phosphorylation of Tau, and endosome abnormalities. Similarly, brain organoid technology can be used to generate patient's-specific tissue that can be used for regeneration and reconstitution after insults such as tumor biopsies, neurodegenerative diseases, and neurological trauma (Hebert and Vijg, 2018 and Ludwig *et al.*, 2018).

Brain organoids have also attracted significant interest in clinical settings, with high applicability for personalized medicine, since in other contexts, organoids have been shown to re-produce personalized clinical outcomes (Vlachogiannis *et al.*, 2018). Therefore, therapeutical testing on patient-derived brain organoids has the potential of reducing the time between patient prognosis and disease progression, which is critical for certain types of cancers, including glioblastoma.

Thus, brain organoid models allow the study of patient pathology *ex vivo*, with results used to improve diagnosis and prognosis, avoiding the current sequential “trial and error” approach, which is often punitive, providing inefficient and non-personalized treatment outcomes. Yet, we are just at the beginning of reaching such an ideal situation due to two major limitations in brain organoid technology. First, organoid models do not recapitulate all cell types within the brain and, most notably, lack functional vasculature. Second, the variability during organoid culture does not allow the systematic assessment of structural mechanisms during the development of brain cytoarchitecture (see, for example, Arora *et al.*, 2017). Without a systematic approach in unpacking the mechanisms behind brain development, we cannot conceive the biophysical and biochemical components that contribute to disease development.

We believe that these limitations could be overcome by applying recently developed novel strategies in chemistry, physics, and materials sciences to create well-controlled 3D environments for brain organoid growth. Novel developments of defined hydrogels can be utilised to engineer predictable organoid formation, allowing for systematic studies of mechanotransduction signalling pathways during brain development, which are frequently overlooked in experiments using animal models (Vining and Mooney, 2017). Finally, the well-controlled 3D environments that new synthetic matrices can offer are essential for the development of platforms suitable for high-throughput screening, which also require compatibility with different optical methods such as fluorescence microscopy.

Therefore, in this review we focus on the role of the tissue microenvironment in cortical brain development and the recent progress on the chemistry of synthetic scaffolds that permit independent control of the tissue biochemical and biophysical microenvironment. Furthermore, we discuss how these scaffolds could be used to optimize the culture of brain organoids and study, in real time, different neural stem cell differentiation and tissue self-organization processes in physiologically relevant settings.

## MECHANICAL PROPERTIES OF THE BRAIN

Physical forces play a critical and interactive role in tissue development, with dysfunctional biomechanics contributing to several brain disorders (Barnes *et al.*, 2017). Tissue stiffness is transduced into biochemical signals through mechanosensors and adhesion molecules located on the surface of cells. These cells specifically interact with the same type of molecule on cell neighbors (e.g., cadherins) or with the ECM (e.g., integrins, Yim and Sheetz, 2012). In particular, ECM stiffness influences different cellular processes including cell proliferation, migration, gene expression (Gurok *et al.*, 2004), stem cell fate specification (Engler *et al.*, 2006; Fu *et al.*, 2010; and Zoldan *et al.*, 2011), cell plasticity, damage response, and regeneration in different brain regions (Dityatev *et al.*, 2010 and Kwok *et al.*, 2011). Moreover, during human brain development, tissue biomechanics profoundly influence interkinetic nuclear migration and orientation of the plane of cell division (Budday *et al.*, 2015). These processes significantly contribute to cortical growth, folding, and the generation of intermediate progenitors (IPs) and basal radial glial cells (RGCs) that form the outer and inner subventricular zones in human brains (Fig. 1).

**FIG. 2.**
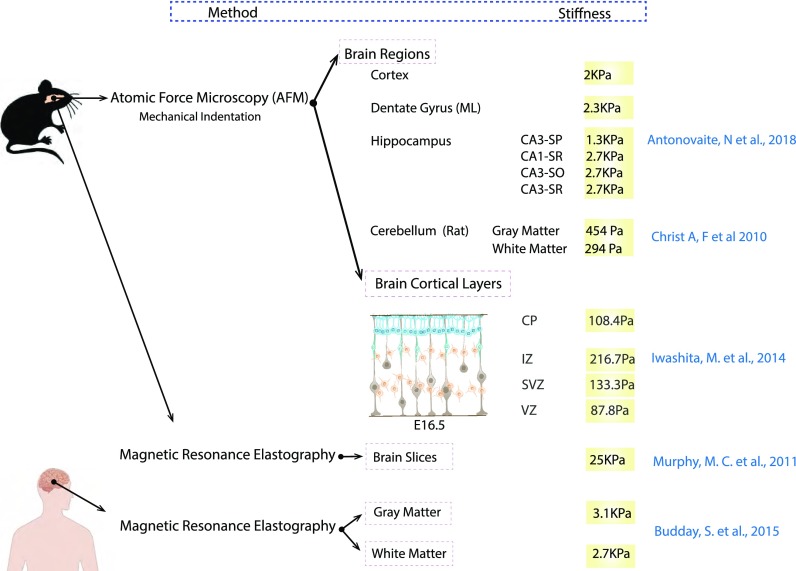
Brain tissue stiffness in mice and human brain regions determined by atomic force microscopy and magnetic resonance imaging. Stiffness values are according to previously reported data (Antonovaite *et al.*, 2018; Budday *et al.*, 2015; Christ *et al.*, 2010; Iwashita *et al.*, 2014; and Murphy *et al.*, 2011) and indicated in the figure.

Yet, the distinctive mechanical properties of the brain tissue, including high viscoelasticity, low stiffness, and heterogeneity, have rendered it one of the most challenging biomaterials to test. Because of this, measurements of brain stiffness have led to highly contrasting results, which appear to be dependent on the measurement technique used and the region of the brain/developmental stage tested (Fig. 2). In particular, during brain development, the mechanical properties of white and grey matter change during gyrification (early at week 22 of gestation). At this stage, white matter is softer than grey matter and after birth becomes similar to the mechanical properties found in mature brains (Budday *et al.*, 2015; Christ *et al.*, 2010; and Finan *et al.*, 2017). Besides brain tissue heterogeneity, the mechanical properties within the brain exhibit non-linear behavior varying markedly between different brain regions such as hippocampus and cortex (Antonovaite *et al.*, 2018). Furthermore, there is a variation within the same brain regions, for example, across different cortical layers, showing a peak in stiffness in the intermediate zone (IZ) and at different embryonic stages (Iwashita *et al.*, 2014). In this particular context, the specific biomechanical properties of the brain tissue can play a key role in determining the number of adult neural stem cells in different cortical regions (Falk *et al.*, 2017). Finally, tissue stiffness has been shown to vary in different neurodegenerative diseases, such as multiple sclerosis (Streitberger *et al.*, 2012) and Alzheimer's diseases (AD, Murphy *et al.*, 2011). In AD patients, Murphy *et al.* showed through magnetic resonance elastography (MRE) that brain tissue becomes significantly softer as a consequence of the altered ECM composition and organization (DuFort *et al.*, 2011; Frantz *et al.*, 2010; and Lu *et al.*, 2012).

## BIOCHEMICAL COMPOSITION OF THE BRAIN ECM: THE BRAIN MATRISOME

The ECM composition offers a sensitive readout for biomechanical changes in biological tissues, particularly in diseases such as cancer (Koh *et al.*, 2018; Mouw *et al.*, 2014). One of the main functions of the ECM is to provide the mechanical and structural support to cells and tissues, constituting the direct microenvironment of the surrounding cells (Freedman *et al.*, 2015). Moreover, the interaction of the ECM with soluble ligands and specific transmembrane receptors provide spatial coordination of the environment and cellular responses. These include the activation of signalling pathways and the organization of the cellular actin cytoskeleton, which contribute to the growth factor's function and the plasma membrane distribution of ion channels and pre/postsynaptic proteins (Sood *et al.*, 2016).

Thus, the brain function relies on the precise ECM composition (detailed in Table I). Moreover, remodelling of the ECM during migration and positioning of cells within the tissue requires disintegrin, metalloproteinase with thrombospondin motif (ADAMT) proteins, and matrix metalloproteinases (MMPs, Zimmermann and Dours-Zimmermann, 2008). Moreover, dysregulation or mutation of ECM components results in a broad range of pathological conditions. For example, mutations in Collagen IV cause cerebral small vessel disease (CSVD), a prevalent neurological disease in aged people associated with stroke, dementia, mood disturbance, and gait problems (Shi and Wardlaw, 2016). Additional changes in proteoglycan (PG) and glycosaminoglycan (GAG) protein expression are related to different neurological disorders such as Alzheimer's disease and schizophrenia (Sethi and Zaia, 2017).

**TABLE I.** Extracellular matrix composition of the human brain according to previously published data (Cui *et al.*, 2013; Rempe *et al.*, 2016; and Schwartz and Domowicz, 2018).

Extracellular matrix brain composition		
Proteoglycans (PG)	Glycosaminoglycans (GAG)	Heparan sulfate (HS)
** **		Chondroitin sulfate (ChS)
** **		Dermatan sulfate (DS)
		Hyaluronan (HA)
		Keratan sulfate (KS)
	Core proteins (Lectican family)	Aggrecan
		Brevican
		Neurocan
		Versican
		Phosphacan
	Growth factors	Epidermal growth factor (EGF)
Glycoproteins (GP)	Tenascin-C	
	Tenascin-R	
	Tenascin-X	
	Reelin	
	Link protein	
	Laminin	
Fibrous protein	Collagen	
	Fibronectin	
	Perineuronal nets (PNNs)	
Metalloproteinases (MMP)	Collagenases	
	Gelatinases	
	Stromelysins	
	Matrilysins	
	Membrane-type	
	Others	

Besides growth factors and MMPs (Dityatev *et al.*, 2010 and Kular *et al.*, 2014), there are other important components found in the ECM, including hyaluronan, glycosaminoglycans (GAG), proteoglycans (PG), and glycoproteins, including tenascin-R and chondroitin sulphate proteoglycans. Furthermore, low levels of fibrous proteins such as collagen, fibronectin, vitronectin, and a neuronal cell surface feature known as perineuronal nets (PNNs) constitute the ECM. Molecules such as aggrecan, brevican, neurocan, versican, and phosphacan show variable levels of expression in different brain regions including the hippocampus, amygdala, and hypothalamus (Dauth *et al.*, 2016). In contrast, molecules such as NCAM, tenascin, and laminin (representing 20% of total brain volume, Anlar *et al.*, 2002) show fluctuating levels of expression during different developmental stages (Sood *et al.*, 2016). Finally, compared with the surrounding non-neurogenic areas, laminins are highly concentrated in the basal lamina and in the ventricular zone (VZ) of the neocortex (Barros *et al.*, 2011). Taken together, the basic structure of the brain ECM is likely to be similar to the proteoglycan component of cartilage (Rauch, 2007), where common ECM “structural” components such as fibronectin and collagen are virtually absent in the adult brain, while proteoglycans are abundantly expressed and are localized to intercellular spaces between neurons and glia (Bonneh-Barkay and Wiley, 2009).

More recently, significant methodological improvements revealed in a detailed quantitative analysis the composition and mechanical properties of tissues (Conway *et al.*, 2017; Mayorca-Guiliani *et al.*, 2017; and Robb *et al.*, 2017). Specifically, work from the Erler lab described the capacity to isolate the ECM, by decellularizing it *in situ*, preserving ECM organization and cytoarchitecture, just as it was in the original tissue (Mayorca-Guiliani *et al.*, 2017). This technique permits the analysis of the exact ECM composition within tissues and inspires possibilities for designing hydrogels for organoid culture with the specific ECM composition (Caiazzo *et al.*, 2016) and organization (Wade *et al.*, 2015) to create the physiological microenvironment of native tissue and more precisely different brain regions.

Thus, it is becoming increasingly clear that the ECM biochemical composition, together with its impact on brain biomechanical properties, are key determinants for the differentiation of different cell types within the brain and the development of brain cytoarchitecture. Yet, current protocols for brain organoid growth use commercially available Matrigel^®^ (Hughes *et al.*, 2010), which poorly reflects the composition of the brain tissue (at any stage) and serves mostly as a scaffold/mechanical support for organoid growth where cells deposit their own ECM (see, for example, Lancaster *et al.*, 2013). Matrigel also presents other limitations including a limited capacity for the user to vary their mechanical properties and biochemical composition to match those of the ECM in the normal brain tissue or even in different brain regions, for example, the cerebral cortex (Lancaster *et al.*, 2013) or cerebellum (Muguruma *et al.*, 2015). These unmatched properties have been shown to have a profound impact on organoid's growth speed, differentiation, and yield production in other contexts (Gjorevski *et al.*, 2016) and being critical for neuronal differentiation, neuronal growth, and development of neural connections in 3D cultures (Sood *et al.*, 2016).

Therefore, the introduction of new synthetic hydrogels, in combination with advances in the neuronal medium composition for optimal culture of functional neurons *in vitro* (Bardy *et al.*, 2015), in brain organoid cultures is likely to provide a better understanding of the ECM's role in neurological disease (Koh *et al.*, 2018). By providing the foundation to inspire new designs of biosynthetic matrices for controlled 3D microenvironments (Arora *et al.*, 2017), this approach will optimize brain organoid growth and further direct the design of high-throughput platforms for personalized drug and genetic screenings. Such developments will offer new opportunities for the advance of new therapeutic treatments for a range of brain diseases.

## SYNTHETIC MATERIALS TO CONTROL THE 3D “HOMOGENOUS” ORGANOID ENVIRONMENT

**FIG. 3.**
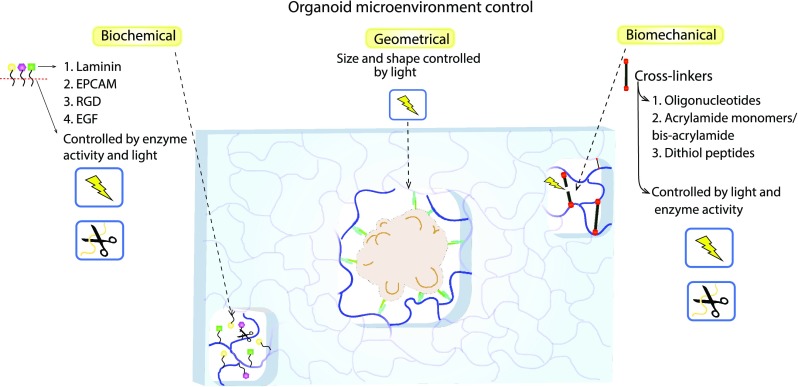
Tunable biomaterials to control the geometrical, biochemical, and biomechanical 3D environment for brain organoid growth. Biomaterials can be functionalized to include different bioactive ligands (normally present in the brain extracellular matrix) and cross-linkers that control the material biomechanical properties. The presence and density of ligands or cross-linkers can be modulated in space and time by light and by the presence of proteases and different microenvironment cues, such as pH or reducing agents.

The synthetic ECM now provides new ways to finely control the physical, chemical, and biological characteristics of the organoid microenvironment (Marti-Figueroa and Ashton, 2017). Considerable progress made in this field highlights the following key advantages: (i) the controlled attachment of biomolecules in the gel structure, (ii) the possibility of modulating the mechanical properties of the materials, and (iii) the degree of degradability of the material (Fig. 3). Although this progress has not yet been applied to brain organoids, such advancements are equally translatable across different organoid models which grow embedded in Matrigel (Broutier *et al.*, 2016; Gjorevski and Lutolf, 2017; Lancaster and Knoblich, 2014a; Morizane and Bonventre, 2017; and Takasato *et al.*, 2016).

Early work for the biocompatible synthetic ECM was performed by Ehrbar *et al.*, who used enzymatically formed and degradable cross-linked polyethylene glycol (PEG)-based hydrogels for the encapsulation and culture of mammalian cells (Ehrbar *et al.*, 2007). In this approach, multi-arm PEGs with vinylsulphone functionality (PEG-VS) are first modified with factor XIIIa substrate peptides (e.g., TG-Gln-RGD) via a Michael-type addition reaction. The cross-linked hydrogel network is then synthesized by mixing the peptide-modified PEGs with thrombin-activated factor XIIIa in the presence of Ca^2+^. More recently, Caiazzo *et al.* used such hydrogels as 3D microenvironments for the generation of iPSCs and reprogramming of tail-tip fibroblasts derived from 4F2A-Oct4-GFP mice (Caiazzo *et al.*, 2016). Using a 3D high-throughput screening approach, the authors systematically studied the effect of hydrogel stiffness, degree of degradability, and biochemical composition (in addition of biomolecules that play a role in regulating pluripotency) in the generation of iPSCs. They demonstrated that cell proliferation was increased with highly degradable gels of stiffness around 300–600 Pa and enriched with laminin or Epcam and the GSK3β inhibitor CHIR99021. The proposed PEG hydrogel presented similar efficiencies (∼45%) in the generation and maintenance of iPSC colonies when compared with traditional Matrigel, of unknown precise chemical composition and high batch-to-batch variability (Caiazzo *et al.*, 2016).

Similar enzymatically formed hydrogels were used for intestinal stem cell expansion and intestinal organoid culture by the Lutolf laboratory (Gjorevski *et al.*, 2016). The authors demonstrated the importance of the hydrogel stiffness on the expansion of intestinal stem cells and organoid formation. While more rigid scaffolds (G′ = 1.3 kPa) promoted the expansion of stem cells as determined by activation of mechano-sensitive yes-associated protein 1 (YAP), softer hydrogels (190 Pa) were more suitable for stem cell differentiation and organoid growth, highlighting the influence of the 3D microenvironment during organoid formation. This was demonstrated by synthesizing materials with dynamic rheological properties. The hydrogels were prepared by incorporating PEG-acrylate monomers within the gel structure in which esters group are more prompt to degradation than the vinylsulfone-functionalized 8-arm PEG (PEG-VS) counterpart. The same group also used similar hydrogels to control the 3D environment of neural morphogenesis (Ranga *et al.*, 2016). In this report, the authors found that synthetic matrices featuring intermediate stiffness (E = 2 and 4 kPa) allowed for the most efficient dorsal-ventral (DV) patterning (Ranga *et al.*, 2016). Using this type of hydrogel in conjunction with a robotic dispensing machine, permitted simultaneous preparation of microarrays >1000 micro-niches to analyze the effect of the independent variation of mechanical properties, biodegradability, cell-ECM, cell-cell interaction proteins, and soluble factors on the proliferation and pluripotency of mouse embryonic stem cells. The approach was applied successfully, providing an in-depth understanding of cell fate in synthetic 3D microenvironments (Caiazzo *et al.*, 2016). Also, the application of hydrogels with variable stiffness and degradability has been shown to influence glioblastoma cell matrix deposition and differently controls glioblastoma mechano-sensitive signaling pathways in 3D cultures (Wang *et al.*, 2014a).

Nguyen *et al.* recently reported an array-based method using synthetic PEG hydrogels for the formation of endothelial networks and screening of angiogenesis inhibitors and the expansion of human embryonic stem cells (hESCs, Nguyen *et al.*, 2017). PEG-based hydrogels were prepared by the well-known thiol-ene click chemistry reaction. Thin hydrogel arrays (120 *μ*m thick) were prepared by photo-polymerization of 20 kDa eight arm PEG-norbornene (PEG-NB), RGD-peptides, dithiol terminated cross-linking peptides, and peptides with affinity for vascular endothelial growth factor (VEGF) compounds. The mechanical properties of the materials were regulated by using different concentrations of the PEG-NB building block. Compared with the naturally derived Matrigel, the prepared hydrogels demonstrated advantages regarding the ability to support vascular network formation (2 times more efficient than Matrigel when using iPSC-endothelial cells) and sensitivity (up to 100% difference) towards the validation of angiogenesis inhibitors. The results shown in this report opened new possibilities for the application of similar synthetic hydrogels for the screening of drugs and other toxic pollutants.

Hydrogels for growing human intestinal organoids were also prepared by a thiol-Michael addition click reaction (Cruz-Acuna *et al.*, 2017). For this, the authors used 4-armed PEG-maleimide (PEG-MAL) monomers of 22 and 44 kDa containing bioactive ligands (e.g., laminin and RGD) that were then cross-linked with dithiol (bis-cysteine) protease-degradable (and non-degradable) peptides. For organoid growth within these hydrogels, spheroids previously formed in Matrigel (or synthetic hydrogel) were encapsulated *in situ* during the hydrogel synthesis, and the organoid's survival and development were systematically evaluated in a range of hydrogels with different mechanical and biochemical properties. High organoid viability was achieved for hydrogels with low polymer density (mechanical properties in the range of those for Matrigel), degradable cross-linkers, and RGD adhesive peptides (Cruz-Acuna *et al.*, 2017). The organoid viability achieved with the optimized materials was comparable to that using Matrigel, whereas organoid formation was strongly dependent on the mechanical properties of the material. The hydrogels were also tested as a delivery vehicle for intestinal organoids to heal colonic wounds. The authors demonstrated the organoid engraftment into the intestinal tissue and improved wound closure as compared with wounds treated with only hydrogel or intestinal organoid without the hydrogel vehicle. At the same time, Goldshmid *et al.* reported the use of the stiffness of PEG-based hydrogels as an effective tool to controlling the proliferation rate and shape of human mesenchymal stem cells [hMSCs (Goldshmid and Seliktar, 2017)]. The gels were prepared by photopolymerization of 10 kDa linear PEG previously modified with fibrinogen and 10 kDa PEG-diacrylate (PEGDA), and hMSCs were encapsulated within the hydrogels during its preparation.

Although the above approaches are useful as they allow site-specific control over where the protein binds to the material, they are limited to the site-specific modification of hydrogels with short peptide sequences (e.g., RGD and laminin peptides). To make static/homogenous synthetic hydrogels with larger proteins, the Griffith lab recently established the reversible site-specific incorporation “post-polymerization” of the human epidermal growth factor (EGF) within hydrogels using a sortase-mediated enzymatic reaction (Cambria *et al.*, 2015). They achieved this by including the LPRTG substrate sequence within the hydrogel and after it was formed added this to the medium sortase and EGF fused to the donor sequence GGG. They found that the EGF incorporated within the gel was biologically active and proportional to the concentration of LPRTG within the gel and the GGG-EGF in the medium. In summary, this approach provides promising results in the development of functionalized synthetic hydrogels having physiologically relevant substrates and ligands.

The above methods of functionalization allow some degree of flexibility on what molecules can be incorporated within these gels however, covalent/site-specific attachment of bio-active molecules could lead to confounding effects on the interpretation of biological responses. Many membrane receptors form part of mechano-sensitive apparatuses that respond differentially to ligands that are freely diffusive or covalently attached to the matrix. To gain insights into the unique properties of cell mechanosensing, a recent report describes the chemical anchorage of bioactive ligands to rings of cyclodextrin that can slide on PEG chains which provide some local diffusivity of ligands within the gel (Tong and Yang, 2016). Using this approach, the Fang lab was able to demonstrate that stem cells alter their behavior in response to the properties of the gel in sliding gels in comparison to non-degradable chemical gels.

## GRADIENT SYNTHETIC MATRICES TO CONTROL THE BIOCHEMICAL AND BIOMECHANICAL 3D ORGANOID ENVIRONMENT

The approaches that we described above only allow for comparison of cell or organoid growth in gels with different and static mechanical properties. This is not suitable for the dynamic analysis of organoid responses to spatial (and temporal, discussed later) changes in their environment which naturally occur in the developing tissues. Such spatial gradients are central in controlling cell proliferation, death, and differentiation (Watt and Huck, 2013) and are determinants for the elongation, folding, and patterning of tissues (Crest *et al.*, 2017). The spatial control of the bio-physicochemical properties was initially achieved by employing photochemical reactions, with initial work mainly driven by the Shoichet and Anseth labs (DeForest *et al.*, 2009; Luo and Shoichet, 2004). In these first descriptions, these labs used different hydrogels (PEG or Agarose) modified with caged chemical substrates that upon irradiation (photolithography or single- or multi-photon lasers) allowed their reactions with biological peptides, patterning the gel. By controlling the power and depth (e.g., multiphoton laser source) of the focal plane, the authors were able to generate gradients of the RGD peptide (Luo and Shoichet, 2004) and produce different types of patterns in 3D environments (DeForest *et al.*, 2009). More interestingly, in this last work, using a thiol-ene photo-coupling chemistry was demonstrated, enabling the patterning of RGD peptides *in situ* and for the first time in the presence of cells (DeForest *et al.*, 2009). Due to the complexity of modifying large proteins with the required chemical compounds for the above reactions, new approaches emerged that allowed the incorporation within hydrogels of large proteins based on protein-protein interactions or enzymatic reactions in a 3D controlled manner (Griffin *et al.*, 2014; Mosiewicz *et al.*, 2013; Wylie *et al.*, 2011). As these constitute general models to precisely modify the biochemistry (i.e., with large proteins) of specific regions within the gel, these will be discussed in more detail in the section on 4D control of the organoid environment.

In addition, non-photochemical approaches for the generation of gel with variable and on-demand chemical gradients have also been reported. The Gerecht lab was able to generate user-defined modular gradient hydrogel constructs based on dual cross-linked bio-functional chemistry (Wei *et al.*, 2017). They designed hydrogels in a way that includes two chemical cross-linking networks, bio-functionalization, and self-healing networks. The latter is a key characteristic to form modular hydrogel constructs with variable gradient distributions and shapes. Self-healing networks are achieved via dynamic Schiff base reaction between N-carboxyethyl chitosan and aldehyde groups of oxidized acrylated hyaluronic acid molecules, which drives the modular gradient units to self-heal, forming a single 3D gel with variable bio-functional and biomechanical properties. Similarly, the Engler and Choi laboratories described an alternative, much simpler method (available in almost any laboratory) to obtain highly reproducible linear gradient polyacrylamide gels in two dimensions (2D, Hadden *et al.*, 2017). In this approach, the authors took advantage of depletion of acrylamide monomers/bis-acrylamide cross-linkers which occurs when the second layer of polyacrylamide is polymerized on top of a ramp of previously polymerized polyacrylamide. Because the mechanical properties of the gel are mainly determined by the initial concentration of acrylamide/bis-acrylamide and the inclination of the ramp, it is possible to produce different gradients of mechanical stiffness which encompass those observed in healthy and diseased tissues. It remains to be tested whether such an approach can be used on hydrogels which allows the embedding of cells in 3D.

## 4D CONTROL OF THE ORGANOID ENVIRONMENT

### Biochemical control

Another aspect that is very important is the ability to modify the bio-physicochemical properties of the organoid environment very precisely in space and time. Studies performed by DeForest *et al.* show the application of click chemistry of substrates susceptible to biocompatible orthogonal photoconjugation and photocleavable reactions; these designer matrices enable the biochemical and biophysical properties of the cell/organoid microenvironment to be varied in 3D and almost instantaneously (DeForest and Anseth, 2011; DeForest *et al.*, 2009; and DeForest and Tirrell, 2015). In particular, these approaches allow high spatiotemporal control of these reactions that are rapidly initialized by delivery of light (i.e., seconds) with very high resolution (∼1 × 1 *μ*m in XY and 3 *μ*m in Z). Currently, the resolution of this approach is limited by the optics of multiphoton microscopy rather than the chemistry of the gels. This has allowed both reversible (DeForest and Tirrell, 2015) and irreversible (DeForest *et al.*, 2009) modifications of the biochemical properties of hydrogels in 3D in the presence of cells and the generation of cavities to control the geometrical environment of cells/organoids (DeForest and Anseth, 2011). These served to analyze either the collective cell migration of cells within channels made up with different biochemical properties or the extraction and analysis of cells within the gel with an unprecedented spatiotemporal resolution. Similar strategies have been utilized to develop materials with customizable vasculature (Arakawa *et al.*, 2017).

Moreover, these principles have been applied for the chemical modification of gels with large proteins or growth factors through protein-protein or enzymatic hydrogel photo-patterning (Mosiewicz *et al.*, 2013 and Wylie *et al.*, 2011). In one of these applications, the Shoichet lab used sequential immobilization of barnase and streptavidin using two-photon unmasking of coumarin-caged thiol modified hydrogels (Wylie *et al.*, 2011). Upon 3D localized irradiation, this allowed the unmasking of thiol groups that were available to react with maleimide-barnase or maleimide-biotin. Posterior and simultaneous incubation of the gel with barstar-sonic hedgehog (barstar-SHH) and streptavidin-ciliary neurotrophic factor (streptavidin-CNTF) allowed independent functionalization in different spatial locations of the gel with these different growth factors and the migration of cells in response to SHH and CNTF gradients. One limitation is that photo-patterning and addition of the biomolecule occurs at different steps, with a washing step in between, limiting the applicability of this approach to *in situ* manipulations of the gel, in the presence of cells or organoids. To overcome this limitation, the Lutolf group developed a similar strategy, in which the bioconjugation step is achieved by an enzymatic ligation reaction, intrinsically compatible with biological systems and which lacks the need of complex chemical conjugation on large biomacromolecules (Mosiewicz *et al.*, 2013). In this approach, PEG gels were modified with a peptide substrate group of activated transglutaminase factor XIII (FXIIIa) caged by a photolabile group. Upon irradiation and in the presence of FXIIIa and an NQEQVSPL-tagged version of VEGF or RGD (also extendable to any protein of interest), the authors were able to generate 3D patterns of biomolecules within the gel, even in the presence of cells (Mosiewicz *et al.*, 2013).

Non-photochemical methods for 4D control of the cell microenvironment have also been achieved but in 2D. The Ashton lab used a combination of micro-patterning and click chemistry to design spatiotemporal regulatable matrices in which they first confine colonies of neuroepithelial stem cells in circles that were not coated with an azide-functionalized poly(ethylene glycol) methacrylate (PEGMA)-grafted substrate (Knight *et al.*, 2015). These PEG brushes are inert and are not cell adhesive. Therefore, cells remain attached and confined to non-PEG regions. By adding the dibenzocyclooctyne (DBCO) conjugated RGD–peptides to the medium, the authors report the functionalization with RGD peptides of the PEG brushes, permitting the expansion and movement of cells outside the initial circular patterns. This showed that such functionalization forms bioactive substrates and promotes the expansion of these islands in a spatiotemporally controlled manner. This constitutes an example of how the chemical composition of the substrates can be controlled with high spatiotemporal resolution although in an irreversible manner. It remains to be tested if such approaches could be extended to 3D by the use of 3D printing and/or bio-printing of cells.

The above methods are suitable for the “addition” of biologically active compounds to the gels. There are also available methods in which already embedded biological compounds can be released on demand from the gel with high spatiotemporal resolution (Ruskowitz and DeForest, 2018). These approaches take advantage of incorporating photolytically degradable cross-linkers or biochemical tethers that upon light (multi-photon) become dissociated from the gel and rapidly change its mechanical properties or release biologically active compounds (Kloxin *et al.*, 2009). In this pioneering work, the Anseth lab used a nitrobenzyl ether-derived moiety incorporated in cross-linkers within PEG hydrogels to derive a photodegradable hydrogel and a nitrobenzyl ether-tethered RGD, able to interact with integrins on human mesenchymal stems cells which were removed with high precision in space and time by two-photon irradiation (Kloxin *et al.*, 2009). More recently, this approach offered more versatility due to the creation of different and orthogonal photocleavable linkers (Azagarsamy and Anseth, 2013) that can also be applied in combination with the above “addition” methods to *reversibly* manipulate the 3D organoid environment, as has been shown in the presence of hMSCs (DeForest and Tirrell, 2015).

### Biomechanical control

Not only the chemistry but also the mechanical properties of gels can be controlled “reversibly” in time as it was recently reported by the Schaffer lab. In this study (Rammensee *et al.*, 2017), the authors aimed to investigate the temporal scales for which “stiffness pulses” were able to define stem cell lineage specification. They achieved this by designing a material on which stiffness could be modulated reversibly without the need to remove the cells from it and on which changes in stiffness did not significantly alter the material's volume (i.e., swelling or contraction). The latter can strain the cells and therefore complicate the interpretation of the results. They used a polyacrylamide ECM system where stiffness can be reversibly manipulated by oligonucleotide based cross-linkers (Rammensee *et al.*, 2017). This approach utilized two distinct side arms of acrydite-functionalized DNA-oligonucleotides that were copolymerized within the polyacrylamide hydrogel. Then, a soluble linker DNA strand was added and hybridized with the two side arms, thus creating additional cross-links that stiffen the gel. The added linker also contained an extra sequence that binds to neither of the sidearms. The subsequent addition of a “release strand,” which fully complements the linker, competitively hybridized the linker, allowing the release of the linker from the gel, softening the gel. Thus, sequential addition of the linker and the release strand allows controlled temporal manipulation of gel stiffness in the physiological relevant 0.3–3 kPa stiff range. Using this approach, the authors were able to determine the temporal window on which neuroepithelial stem cells are mechanosensitive (<36 h). Within this period, if a pulse of a stiff substrate is applied, the commitment to neuronal differentiation is abolished, a result that could be mimicked by overexpression of YAP (Rammensee *et al.*, 2017). Although the methods offer temporal control of gel mechanics, it does this in a semi-slow manner and without spatial control. Recent efforts have sought to address these limitations through the use of materials containing on photoresponsive proteins whose moduli can be reversibly cycled with spatiotemporal control in the presence of living cells (Liu *et al.*, 2018).

The above methods only permit the spatiotemporal control of gels based on one or two environmental clues, such as the presence of light and/or enzymatic activity. To circumvent this limitation, and increase design flexibility to the system, the DeForest lab recently developed a modular design approach (Badeau *et al.*, 2018). Here, a user-programmed Boolean-logic algorithm is used to impart hydrogels with precise degradative responsiveness using multiple environmental clues to control its biomechanical properties in space and time (Badeau *et al.*, 2018). The information governing the environmental responsiveness of the hydrogel is embedded within the cross-linker domain, which could be sensitive to specified combinations of light, enzymatic reactions, and/or redox conditions of the environment. By designing different cross-linkers and introducing these degradative units in series or in parallel, the authors first were able to design logic YES/OR/AND gates that control the biomechanical properties of gels based on the combination of these three environmental cues. The hierarchical combination of these gates allowed the authors to obtain 17 different gel types, all with similar properties but unique in their responsivity to the precise combination of redox, metalloproteinase (MMP) activity, and light. The approach permitted the precise release of cytotoxic drugs to clues that mimic the tumor microenvironment in tissues and the release of cells from the gel in a very specific manner (Badeau *et al.*, 2018). In the future, such approaches will be useful to generate 3D biomechanical gradients to control stem cell differentiation and brain organoid formation and vascularization (Pham *et al.*, 2018) and the interaction between different types of organoids.

While the literature discussed above used non-porous synthetic hydrogels as a substitute to Matrigel, the application of porous polymers (Arrua *et al.*, 2013; Arrua *et al.*, 2014; and Khodabandeh *et al.*, 2017) might be more advantageous in contrast to non-porous matrices by, for example, increasing the control over the scaffold's surface topology, surface chemistry, porosity, and space confinement. Porous materials could facilitate diffusion processes where chemicals from cell culture media (e.g., growth factors) could easily diffuse through the 3D network and access the organoids that are physically confined in space. Even though soft porous polymers have not been used for organoid growth, their use as scaffolds for cells (Aliperta *et al.*, 2017) and spheroid (Tam *et al.*, 2016) culture anticipates their application in the organoid field.

## SYNTHETIC MATERIALS TO CONTROL THE “GEOMETRY” OF THE 3D CELL MICROENVIRONMENT

One of the advantages of synthetic hydrogels is their capacity to be prepared with different geometries by using microfabrication techniques. This allows the application of synthetic materials with well-defined 3D architectures that mimic the microenvironments where cells develop and differentiate. Early work by the Anseth lab has shown the creation of arrays of culture wells of varying sizes and shapes which were patterned into hydrogels using lithography. Moreover, by using hydrogels composed of photolabile and enzyme-labile cross-links and pendant adhesion sequences, they were able to pattern, in a spatially controlled manner, the hydrogel with light and permit cell-dictated microenvironment remodeling through enzyme secretion (Kloxin *et al.*, 2009). Using this approach, the authors were able to investigate the role of geometry on the differentiation of primary mouse alveolar cells using immunofluorescence and connect well-separated clusters by eroding channels between wells using multiphoton microscopy (Kloxin *et al.*, 2012). More recently, Bao and co-workers also demonstrated how the geometry of 3D microniches affects the single cell function. They photo-polymerized hyaluronic acid-based 3D hydrogels microniches to control the size and volume of mesenchymal stem cells (MSCs) and demonstrated how these variables affected the MSC function and fate (Bao *et al.*, 2017). Similar types of hydrogels were also recently patterned with microfluidic channels (using laser photo-ablation) during cell culture experiments (Brandenberg and Lutolf, 2016). Micro-channels with a variety of shapes could be prepared *in situ* within PEG hydrogels and used to deliver signaling molecules that can diffuse and reach the cultured cell/organoids. Moreover, a combination of photo-patterning of PEG hydrogels using stereo-lithography on porous filters allowed the creation of perfused 3D culture platforms for the growth of hepatocytes (Neiman *et al.*, 2015). More recently, growth on organoids sandwiched between polydimethylsiloxane (PDMS) and semi-permeable polycarbonate membranes allowed the growth of “flat” brain organoids that can be imaged for weeks and which were used to understand the mechanics of cortical folding and how is it affected in brain organoids derived from iPSC cells from patients with Lissencephaly (Karzbrun *et al.*, 2018). Finally, several reports by the Qin lab with the implementation of different brain-organoids on a chip for high-throughput applications have been reported (Wang *et al.*, 2018a; Wang *et al.*, 2018b; Yin *et al.*, 2018; Zhu *et al.*, 2017a; and Zhu *et al.*, 2017b). It is foreseen that these approaches will permit a better understanding of how the geometrical constraints of the microenvironment control brain tissue architecture and the specification of different brain regions.

## FUTURE DIRECTIONS

Substantial differences between human and experimental models (mice, fly, zebrafish, and 2D culture of cell lines) make it very difficult to investigate central nervous system diseases, particularly for therapeutic applications. Brain organoids now provide a novel experimental platform that allows recapitulation of basic brain structural organization and its cellular composition and biomechanical properties. Recent advances in the design of biomaterials with properties that can be controlled in space and time will address the current limitations in brain organoid technology and therefore has strong medical implications.

Specifically, the use of substrates with well-known biochemical and biophysical compositions and geometrically defined constraints can reduce variability in the culture of region-specific brain organoids. Reproducibility in the developmental time-frame for fully differentiated organoids, as well as in the morphology/architecture, is important to quantitatively define subtle differences between organoids derived from different patients. The complementation of well-controlled microenvironments with customizable microfluidic chambers using photo-degradation or filter approaches (as discussed above) will overcome the requirement for constant agitation—needed for proper oxygenation, which prevents early death of the organoid—to produce fully differentiated organoids (Lancaster *et al.*, 2013; Lindborg *et al.*, 2016; and Qian *et al.*, 2016). Removal of agitation requirements will also expand the use of brain organoid technology to visualize in real time and *in situ* the development of different cortical regions and to high-throughput applications using fluorescence microscopy (either multiphoton or light sheet), which are currently limited to architecturally simple intestinal organoids (Gracz *et al.*, 2015; Gunasekara *et al.*, 2018; Wang *et al.*, 2013; and Wang *et al.*, 2014b). Moreover, it will also be possible to combine the above with immunofluorescence and different clearing methods already developed for brain tissues (Ke *et al.*, 2016; Li *et al.*, 2017; Silvestri *et al.*, 2016; and Susaki and Ueda, 2016). Clearing methods allow deeper penetration of light and cause less optical aberrations by matching the refractive index of the mounting media and thus suitable for the analysis of brain tissue morphometry and cellular architecture (Ke *et al.*, 2016 and Li *et al.*, 2017).

The ability to manipulate the mechanical environment of organoids will allow researchers also identify different mechanotransduction pathways involved in brain morphogenesis and disease, permitting the analysis of their activation and function in real time within living brain tissue (i.e., by using different types of genetically encoded FRET reporters (Acharya *et al.*, 2017; Cameron *et al.*, 2014; Lagendijk *et al.*, 2017; Priya *et al.*, 2015; and Ratheesh *et al.*, 2012). For example, there is a significant body of literature which points out mutations or changes in gene expression of different GTPases (Rac, Cdc42, and RhoA) and their upstream regulators/downstream effectors in the development of different brain disorders (Cappello, 2013; Cappello *et al.*, 2006; Cappello *et al.*, 2012; Huang *et al.*, 2017; Li *et al.*, 2016; Lin *et al.*, 2015; Pan *et al.*, 2015; Ravindran *et al.*, 2017; Reijnders *et al.*, 2017; and Santana and Marzolo, 2017) and in glioblastoma cell invasion (Arden *et al.*, 2015; Cui *et al.*, 2017; Liu *et al.*, 2015; Shah *et al.*, 2016; Shi *et al.*, 2017; and Wong *et al.*, 2015); however, it remains unclear how these proteins contribute to diseases. We believe that the combination of synthetic materials, optical imaging, and brain organoid technology will shed light on these processes.

Finally, the recent development in bio-printing and controlled co-culture of different cell types (Zhuang *et al.*, 2018), in combination with brain organoid growth on synthetic matrices engineered with microcavities and microfluidic devices (Wang *et al.*, 2018a; Wang *et al.*, 2018b; and Yin *et al.*, 2018), presents promising future studies of the interactions of organoids with other different cell types, such as immune/endothelial cells (i.e., to promote organoid vascularization, Pham *et al.*, 2018). In conclusion, these developments will permit—in a dish—a model that closely mimics the real complexity of the native brain tissue and create opportunity for real progress in the development of new therapeutic treatments for brain diseases.
